# Pleiotropic effects of fenofibrate therapy on rats with hypertriglycemia

**DOI:** 10.1186/s12944-015-0032-3

**Published:** 2015-04-14

**Authors:** Bing Sun, Yuan Xie, Jinfa Jiang, Yiping Wang, Xiaolin Xu, Cuimei Zhao, Feifei Huang

**Affiliations:** Department of Cardiology, Tongji Hospital, Tongji University, Shanghai, 200092 China

**Keywords:** Fibrate, Hypertriglycemia, Pleiotropic effects

## Abstract

**Background:**

Cardio-protective effect of fibrate therapy is controversial and current research is to evaluate the effect of fenofibrate therapy on rats with hypertriglycemia.

**Methods:**

Rats with hypertriglycemia were produced by 10% fructose administration (10 ml daily) for 4 weeks. After model has been successfully produced as reflected by increased triglyceride level, different doses of fenofibrate, namely low dose (50 mg/kg body weight) and high dose (100 mg/kg body weight), were orally prescribed for 2 weeks. At baseline, 4 weeks of fructose administration and 2 weeks of fenofibrate therapy, parameters of interest were evaluated and compared.

**Results:**

At baseline, no significant differences of parameter were observed between groups. After 4 weeks of fructose prescription, triglyceride level increased in company with high density lipoprotein cholesterol (HDL-C) and apoprotein A1 (ApoA1) decreased. C-reactive protein (CRP) and malondialdehyde (MDA) levels were also elevated. Endothelial function was impaired as reflected by reduced nitric oxide (NO) production and elevated serum asymmetric dimethylarginine (ADMA) level. All these changes were significant as compared to the control group (*P* < 0.05), suggesting that short-term of triglyceride elevation could potently initiate atherosclerosis. With 2 weeks of fenofibrate therapy, in comparison to un-treated group, triglyceride level was significantly reduced in parallel with HDL-C and ApoA1 elevation. Inflammation and oxidation were also profoundly ameliorated as reflected by CRP and MDA reduction. Notably, NO production was enhanced in company with serum ADMA level decrease. Overall, these improvements manifested in a dose-dependent manner, which was supported by multivariate regression analysis showing that after adjusted to other variables, the dose of fenofibrate therapy remained significantly associated with NO production and serum ADMA level, with OR of 1.042 (high-dose versus low-dose, 95% CI 1.028-1.055, P < 0.05).

**Conclusions:**

Fenofibrate therapy not only could reduce triglyceride level but also confer pleiotropic effects in terms of endothelium-protection and amelioration of inflammation and oxidation in a dose-dependent manner.

## Introduction

Atherosclerotic cardiovascular disease (ASCVD) is the leading cause of morbidity and mortality worldwide [1]. Dyslipidemia, predominantly featured by increased low density lipoprotein cholesterol (LDL-C) level, is one of the most important risk factors for ASCVD [2]. Therefore, in the past decades, LDL-C has become the principal therapeutic target for reducing the incidence and prevalence of ASCVD [3,4]. Nonetheless, despite target LDL-C level has been achieved by statins therapy, residual cardiovascular risk still persists [5]. Recently, two large clinical researches show that decreased fasting or non-fasting triglyceride levels due to APOC3 gene loss-of-function mutation is significantly associated with reduced ischemic cardiovascular events, which is independent of LDL-C reduction [6,7]. Therefore, it is reasonable to postulate that increased triglyceride level may at least partially contribute to the residual cardiovascular risk, if not at all [8]. However, whether there is a causal relationship between triglyceride reduction and cardiovascular events reduction is still inconclusive and the underlying mechanisms are far from clearly understood yet [9-11]. Previously, many epidemiological studies show that no consistent cardio-protective effects could be obtained with triglyceride reduction by fibrate therapy [5,12,13], a medicine specific for triglyceride regulation.

With regard to the conflicting findings of previous researches as well as recent emerging evidence suggesting that reduced triglyceride level due to loss-of-function mutation of APOC3 gene is associated with reduced ischemic cardiovascular risk, we conducted a basic preliminary research using hypertriglycemia animal model in order to investigate the relationship between triglyceride and endothelial dysfunction, a hallmark of early stage of atherosclerosis. In addition, the efficacy of fibrate on triglyceride and endothelial function will also be evaluated. We believed that results from our present study would provide insights for better understanding the effects of triglyceride on atherosclerosis and ASCVD development.

## Methods

### Animal preparation and research protocol

Forty male Sprague–Dawley (SD) rats weighing 200–220 g were used in present research, and studied protocol was approved by the Ethic Committee of Tongji Hospital, Tongji University, Shanghai. Briefly, 30 rats were randomly selected for hypertriglycemia model establishment by virtue of orally prescribing 10% fructose solution (10 ml per day) for totally 4 weeks. The other 10 rats used as the control group (CONT) were given normal saline (10 ml per day) for 4 weeks, too. Thereafter, serum level of triglyceride was assessed to confirm model successfully produced. Rats with hypertriglycemia were then randomly and evenly assigned into 3 groups as follows: low fenofibrate (LFF) group (50 mg/kg body weight, p.o.), high fenofibrate (HFF) group (100 mg/kg body weight, p.o.) and non-treated (NT) group. The duration of medical therapy was 2 weeks, and thereafter, serum levels of lipid profiles and other parameters of interest were evaluated.

### Laboratory examination

At baseline and with 4 weeks of fructose administration, fasting venous blood was collected for parameters of interest evaluation. Biochemical parameters such as lipid profiles including triglyceride (TG), LDL-C, high density lipoprotein cholesterol (HDL-C), total cholesterol (TC), apoprotein A1 (ApoA1) and apoprotein B (ApoB), fasting blood glucose (FBG) and liver enzymes involving alanine aminotransferase (ALT) and aspartase aminotransferase (AST) were concomitantly assessed by Automatic Biochemistry Analyzer (Beckman coulter UniCel DxC 800 Synchron). Serum levels of inflammatory and oxidative markers such as C-reactive protein (CRP assay Kit, Nanjin Jianchen Bioengineering Institute, H151) and malondialdehyde (MDA assay Kit, Nanjin Jianchen Bioengineering Institute, A003-2) were assessed in accordance to the manufacture’s instruction. Indicators of endothelial function including nitric oxide (NO) production (Total Nitric Oxide Kit, Beyotime, Haimen, China, S0023) and serum level of asymmetric dimethylarginine (ADMA, enzyme-linking immune-absorbent assay kit, Shanghai Westan Bio-technology Company) were also evaluated. After 2 weeks of fenofibrate therapy, parameters as mentioned above were detected so as to define the effects of fenofibrate therapy on hypertriglycemia, endothelial function and inflammation and oxidation.

### Statistical analyses

All continuous variables were expressed as mean ± SD, and analyses were performed with SPSS software, version 19.0 (SPSS Science, Chicago, IL, USA). Comparison between each group was conducted by the Student’s t-test when the data was normally distributed, otherwise was compared by Wilcoxon rank-sum test. Multivariate regression analysis was also conducted to evaluate the relationship between the dose of fenofibrate and the changes of endothelial function, and a value of *P* < 0.05 was considered statistically significant.

## Results

### Parameters comparisons prior- and post-model establishment

In order to evaluate the changes of lipid profiles, endothelial functions and biomarkers of inflammation and oxidation prior- and post-model establishment, serum levels at baseline and after 4 weeks of fructose administration were drawn. As shown in Table 1, parameters of interest at baseline between each group were comparable. With 4 weeks of fructose administration, serum level of triglyceride was significantly increased in company with decreased HDL-C and ApoA1 levels as compared to the control group (*P* < 0.05). No prominent changes of other lipids such as TC, LDL-C, and ApoB were observed between groups. Serum levels of CRP and MDA were profoundly elevated in parallel with triglyceride elevation, suggesting that short-term of hypertriglycemia had robust effects on initiating systemic inflammation and oxidation. Two sensitive and specific biomarkers of endothelial function, named NO and ADMA, correspondingly reduced in accordance to triglyceride elevation as presented in Table 1, strongly indicating that hypertriglycemia, independent of LDL-C elevation, contributed to endothelial dysfunction. No significant changes of FBG, ALT and AST were observed between groups.

**Table 1:** **Parameters comparisons prior- and post-model establishment**

**Variables**	**CONT**	**UT**	**LFF**	**HFF**
**Baseline**				
**TG (mmol/L)**	0.98 ± 0.10	0.96 ± 0.11	0.96 ± 0.12	0.99 ± 0.11
**TC (mmol/L)**	3.26 ± 0.17	3.27 ± 0.20	3.24 ± 0.14	3.27 ± 0.19
**LDL-C (mmol/L)**	1.99 ± 0.13	1.98 ± 0.12	1.99 ± 0.14	2.00 ± 0.16
**HDL-C (mmol/L)**	1.04 ± 0.05	1.05 ± 0.03	1.05 ± 0.04	1.05 ± 0.04
**ApoA1 (mmol/L)**	1.00 ± 0.03	1.00 ± 0.04	1.01 ± 0.02	1.01 ± 0.03
**ApoB (mmol/L)**	0.94 ± 0.05	0.94 ± 0.06	0.96 ± 0.03	0.95 ± 0.02
**ALT (U/L)**	28.5 ± 6.2	27.2 ± 3.9	25.8 ± 3.0	27.0 ± 2.3
**AST (U/L)**	29.6 ± 4.0	28.5 ± 3.7	27.3 ± 4.1	27.8 ± 4.0
**FBG (mmol/L)**	5.78 ± 0.13	5.85 ± 0.13	5.69 ± 0.14	5.90 ± 0.14
**CRP (mg/L)**	1.75 ± 0.23	1.77 ± 0.16	1.80 ± 0.15	1.75 ± 0.16
**MDA(nmol/L)**	0.98 ± 0.13	0.96 ± 0.10	0.99 ± 0.15	0.97 ± 0.14
**NO (μmol/L)**	14.16 ± 1.22	13.54 ± 1.09	13.87 ± 1.13	13.99 ± 1.15
**ADMA (nmol/L)**	69.24 ± 10.17	68.30 ± 10.28	67.15 ± 11.52	68.64 ± 12.08
**4 weeks later**				
**TG (mmol/L)**	0.97 ± 0.12*	2.26 ± 0.36	2.29 ± 0.27	2.28 ± 0.20
**TC (mmol/L)**	3.28 ± 0.31	3.43 ± 0.41	3.40 ± 0.43	3.37 ± 0.38
**LDL-C (mmol/L)**	1.99 ± 0.15	2.02 ± 0.26	2.05 ± 0.33	2. 03 ± 0.22
**HDL-C (mmol/L)**	1.06 ± 0.05*	0.91 ± 0.03	0.90 ± 0.05	0.91 ± 0.03
**ApoA1 (mmol/L)**	1.02 ± 0.03*	0.86 ± 0.05	0.86 ± 0.04	0.88 ± 0.05
**ApoB (mmol/L)**	0.95 ± 0.04	0.98 ± 0.04	0.97 ± 0.03	0.98 ± 0.04
**ALT (U/L)**	26.7 ± 5.2	31.5 ± 4.2	30.6 ± 3.7	32.1 ± 4.4
**AST (U/L)**	28.7 ± 4.2	32.1 ± 3.0	32.0 ± 3.6	33.4 ± 4.2
**FBG (mmol/L)**	5.89 ± 0.16	6.04 ± 0.16	6.01 ± 0.10	6.03 ± 0.12
**CRP (mg/L)**	1.76 ± 0.11*	9.15 ± 1.07	9.39 ± 1.06	9.27 ± 1.06
**MDA(nmol/L)**	0.98 ± 0.14*	3.36 ± 0.40	3.47 ± 0.36	3.40 ± 0.47
**NO (μmol/L)**	13.87 ± 1.14*	9.24 ± 1.06	9.29 ± 1.04	9.37 ± 1.05
**ADMA (nmol/L)**	69.32 ± 11.31*	96.45 ± 13.35	99.36 ± 12.20	100.24 ± 11.61

Denote: *P < 0.05 versus other groups.

### Parameters comparisons with different doses of fenofibrate therapy

In order to assess the effects of fenofribate therapy on hypertriglycemia, endothelial function and systemic inflammation and oxidation, different doses of fenofibrate, named low- and high-dose (LFF and HFF), were prescribed for 2 weeks. As presented in Table 2, with 2 weeks of fenofibrate therapy, as compared to the un-treated (UT) group, serum levels of TG, HDL-C and ApoA1 were improved in both LFF and HFF groups. Serum levels of CRP and MDA were gradually decreased in correspondence to the doses of fenofibrate therapy. Notably, the improvements of NO production and serum ADMA level were also presented in a dose-dependent manner. All above improvements suggested that the efficacy of fenofibrate therapy on hypertriglycemia, endothelial function, systemic inflammation and oxidation was associated with the dose used.

**Table 2:** **Parameters comparisons with different doses of fenofibrate therapy**

**Variables**	**CONT**	**UT**	**LFF**	**HFF**
**TG (mmol/L)**	0.95 ± 0.06*	2.30 ± 0.40	2.18 ± 0.35	1.96 ± 0.24#
**TC (mmol/L)**	3.27 ± 0.25	3.31 ± 0.36	3.30 ± 0.24	3.28 ± 0.16
**LDL-C (mmol/L)**	1.99 ± 0.13	2.04 ± 0.16	2.01 ± 0.24	2.00 ± 0.21
**HDL-C (mmol/L)**	1.06 ± 0.04	0.90 ± 0.04	0.97 ± 0.05	1.03 ± 0.03#
**ApoA1 (mmol/L)**	1.03 ± 0.03	0.86 ± 0.04	0.92 ± 0.02	0.99 ± 0.05#
**ApoB (mmol/L)**	0.96 ± 0.05	0.98 ± 0.03	0.98 ± 0.04	0.98 ± 0.02
**ALT (U/L)**	27.5 ± 2.9	30.2 ± 1.6	29.0 ± 2.2	28.2 ± 1.5
**AST (U/L)**	28.4 ± 2.3	31.0 ± 2.5	29.5 ± 2.4	28.6 ± 1.0
**FBG (mmol/L)**	5.88 ± 0.15	6.04 ± 0.14	6.00 ± 0.13	6.00 ± 0.10
**CRP (mg/L)**	1.80 ± 0.13*	9.20 ± 1.04	9.09 ± 0.98	8.92 ± 0.76#
**MDA(nmol/L)**	0.98 ± 0.11*	3.35 ± 0.13	3.21 ± 0.16	3.03 ± 0.12#
**NO(μmol/L)**	13.21 ± 1.03*	9.16 ± 1.12	9.35 ± 1.08	9.98 ± 1.00#
**ADMA (nmol/L)**	68.24 ± 9.13*	97.33 ± 10.72	95.46 ± 10.33	91.25 ± 11.25#

Denote: *P < 0.05 versus other groups, ^#^ *P* < 0.05 versus the CONT group.

### Relationship between the dose of fenofibrate and the change of endothelial function

In order to investigate the relationship between the dose of fenofibrate use and the changes of endothelial function, multivariate regression analysis was performed. After adjusted to TC, LDL-C, HDL-C, TG, FBG, CRP and MDA, the dose of fenofibrate therapy remained significantly associated with NO production and serum ADMA level, with OR of 1.042 (high-dose group versus low-dose group, 95% CI 1.028-1.055, P < 0.05).

## Discussion

The effects of hypertriglycemia on atherosclerosis and ASCVD have been a controversial issue for decades [8,11]. Previously, some basic studies and observational clinical researches show that increased triglyceride level contributes to the initiation and progression of atherosclerosis [12,14,15]. Nonetheless, large and randomized clinical trials using fibrate, the potent triglyceride-modulation medicine, show no consistent clinical benefits on reducing cardiovascular events in spite of profound reduction of triglyceride level [16,17]. Recently, two large clinical trials demonstrate that decreased fasting or non-fasting triglyceride level, owing to loss-of-function mutation of APOC3 gene, is independently associated with reduced ischemic cardiovascular risk [6,7]. Of clinical relevance, our present research also shows that as compared to the non-treated group, triglyceride reduction by fenofibrate therapy results in the improvement of endothelial function and systemic inflammation and oxidation, and these changes are manifested in a dose-dependent manner, suggesting that in the early stage of atherosclerosis initiating by hypertriglycemia, fenofibrate therapy may play a role on vascular-protection.

Knowingly, LDL-C is the so-called bad cholesterol which leads to prevalent atherosclerosis and ASCVD. Statins, a specific cholesterol-modulation medicine, potently decreases LDL-C level and confers robust cardio-protective effects. However, despite LDL-C level has been reduced to less than 1.8 mmol/dL, a substantial number of patients still have recurrent cardiovascular events which now recognized as residual cardiovascular risks [18,19]. Many risk factors, such as lipoprotein (a) and lipoprotein-associated phospholipase A2, have been identified responsible for the residual cardiovascular risks as suggested by previous observational researches [20-24]. Whether increased triglyceride level is accounted for the residual cardiovascular risks is unclear yet, however. Evidence from two recent large clinical researches suggested that the whole-life low triglyceride level due to loss-of-function mutation of APOC3 gene was independently associated with low cardiovascular risk. While previous epidemiological researches using fibrate temporarily decreasing triglyceride level resulted in contradictory outcomes. To our best knowledge, it might be at least partially due to the following two mechanisms. In the first place, the effect of whole-life low triglyceride level on cardiovascular system was strikingly and mechanically different from the transient low triglyceride level derived from fibrate therapy. On the other hand, benefits resulted from loss-of-function mutation of APOC3 gene might not only ascribe to whole-life low triglyceride level but might also be associated with other unexplored mechanisms.

In order to reveal the effects of short-term hypertriglycemia on atherosclerosis, as reflected on endothelial dysfunction, and to evaluate the efficacies of fenofibrate treatment on endothelial function in the setting of hypertriglycemia, we conducted a preliminary basic research. Results from our present research showed that short-term of hypertriglycemia, produced by fructose administration, rendered robust adverse effects on endothelium in rats, as reflected by decreased NO production and increased serum ADMA level. Mechanically [25,26], fasting triglycerides are markers of remnant particles including very low density lipoprotein cholesterol and intermediate density lipoprotein cholesterol. Like LDL-C, these lipoprotein-rich triglycerides are potential to penetrate endothelial barriers, retain in arterial intima and cause atherosclerosis. Additionally, promoting systemic inflammation and oxidation owing to hypertriglycemia also accelerates atherosclerosis. Therefore, preliminary data from our present research supported the notion that increased triglyceride level was at least partially accounted for the residual cardiovascular risks, if not completely.

We then evaluated the efficacies of different doses of fenofibrate therapy on hypertriglycemia, endothelial function, and systemic inflammation and oxidation. As presented in Table 2, 2 weeks of fenofibrate therapy provided benefits in terms of triglyceride-reduction, increased NO production and decreased ADMA level, as well as decline of CRP and MDA levels. Overall, these improvements manifested a dose-dependent manner which was further supported from results by multivariate regression analysis. After adjusted to lipid profiles, CRP and MDA, the dose of fenofibrate therapy remained significantly associated with NO production and serum ADMA level, with OR of 1.042 (high-dose group versus low-dose group, 95% CI 1.028-1.055, P < 0.05). Regarding the adverse effects of hypertriglycemia on endothelium, we considered that triglyceride reduction might be the source of the upcoming benefits in terms of prominent improvement of endothelium and systemic inflammation and oxidation. Secondly, we observed that with fenofibrate therapy, serum levels of HDL-C and ApoA1 simultaneously elevated. As is well known that, HDL-C is the good cholesterol which can transport cholesterol from periphery to liver and then excreting into intestinal tract [27]. Therefore, we considered that increased HDL-C and ApoA1 levels might also be the underlying mechanism associated with the benefits derived from fenofibrate therapy. Intriguingly and importantly, since over-consumption of fat or carbohydrate as animal-model established in our present research is the main cause of hypertriglycemia, it is possible that adding nutraceuticals or functional food ingredients into fenofibrate therapy could confer synergistic benefits on vascular system [28]. With respect to the safety of fenofibrate therapy, we further evaluated the liver enzymes which showed no significant changes of ALT and AST after two weeks’ therapy between groups.

## Conclusions

Results from our present preliminary research suggest that increased triglyceride level may confer residual cardiovascular risk mechanically, and fenofibrate therapy not only could reduce triglyceride level but also could confer pleiotropic effects in terms of endothelium-protection and amelioration of systemic inflammation and oxidation with a dose-dependent manner.
